# Prior choice and data requirements of Bayesian multivariate hierarchical models fit to tag‐recovery data: The need for power analyses

**DOI:** 10.1002/ece3.9847

**Published:** 2023-03-26

**Authors:** Cody E. Deane, Lindsay G. Carlson, Curry J. Cunningham, Pat Doak, Knut Kielland, Greg A. Breed

**Affiliations:** ^1^ Department of Biology and Wildlife Fairbanks Alaska USA; ^2^ Department of Biology, University of Saskatchewan Saskatchewan Saskatoon Canada; ^3^ College of Fisheries and Ocean Sciences Juneau Alaska USA; ^4^ Department of Biology and Wildlife and Institute of Arctic Biology University of Alaska Fairbanks Fairbanks Alaska USA

**Keywords:** Bayesian analysis, capture–recapture, harvest assessment, hierarchical models, multivariate normal distribution, random effects

## Abstract

Recent empirical studies have quantified correlation between survival and recovery by estimating these parameters as correlated random effects with hierarchical Bayesian multivariate models fit to tag‐recovery data. In these applications, increasingly negative correlation between survival and recovery has been interpreted as evidence for increasingly additive harvest mortality. The power of these hierarchal models to detect nonzero correlations has rarely been evaluated, and these few studies have not focused on tag‐recovery data, which is a common data type. We assessed the power of multivariate hierarchical models to detect negative correlation between annual survival and recovery. Using three priors for multivariate normal distributions, we fit hierarchical effects models to a mallard (*Anas platyrhychos*) tag‐recovery data set and to simulated data with sample sizes corresponding to different levels of monitoring intensity. We also demonstrate more robust summary statistics for tag‐recovery data sets than total individuals tagged. Different priors led to substantially different estimates of correlation from the mallard data. Our power analysis of simulated data indicated most prior distribution and sample size combinations could not estimate strongly negative correlation with useful precision or accuracy. Many correlation estimates spanned the available parameter space (−1,1) and underestimated the magnitude of negative correlation. Only one prior combined with our most intensive monitoring scenario provided reliable results. Underestimating the magnitude of correlation coincided with overestimating the variability of annual survival, but not annual recovery. The inadequacy of prior distributions and sample size combinations previously assumed adequate for obtaining robust inference from tag‐recovery data represents a concern in the application of Bayesian hierarchical models to tag‐recovery data. Our analysis approach provides a means for examining prior influence and sample size on hierarchical models fit to capture–recapture data while emphasizing transferability of results between empirical and simulation studies.

## Graphical abstract

We assessed the power of hierarchical Bayesian multivariate models to detect negative correlation between survival and recovery probability when these parameters are estimated as correlated random effects from tag recovery data. We found inference depended on prior choice when we fit our models to empirical mallard data. A power analysis of simulated data revealed that most prior and sample size combinations we evaluated could not recover known correlation values.

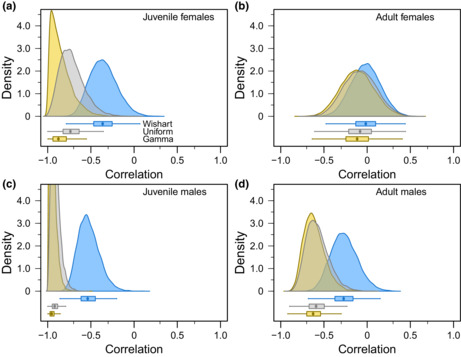

## 1 INTRODUCTION

Modern approaches to quantifying relationships among demographic parameters include modeling temporal variation in vital rates as correlated random effects drawn from multivariate normal distributions (Fay et al., 2021; Link & Barker, 2005; Riecke et al., 2019). This approach allows the correlation between parameters to be estimated without bias from sampling covariation between parameters (Otis & White, 2004). Bayesian modeling frameworks offer a more tractable approach to fitting relatively complex random effects structures when compared to Frequentist approaches, thereby Bayesian estimation is a natural choice when fitting multivariate hierarchical models (Royle & Link, 2002). While Bayesian estimation is advantageous when fitting complex random effects structures, Bayesian inference includes the influence of prior distributions on posterior distributions (Gelman et al., 2014).

In both conservation and management settings, demographic observations are often obtained through capture–recapture methods (Williams et al., 2002). Multivariate hierarchical models specific to capture–recapture data have estimated correlations between survival and recruitment (Link & Barker, 2005), juvenile and adult survival (Riecke et al., 2019), survival and reproduction (Paterson et al., 2018), reproductive effort and reproductive frequency (Badger et al., 2020), and parameters estimated with integrated population models (Koons et al., 2017; Schaub et al., 2013). Additionally, some studies have focused on quantifying the impact of harvest on vital rates like annual survival (or natural mortality) by estimating correlation between random effects from tag‐recovery data, which is a capture–recapture data type in which individuals are reencountered after experiencing mortality (Arnold et al., 2016; Bartzen & Dufour, 2017; Koons et al., 2014; Servanty et al., 2010). Tag‐recovery data are advantageous in that the harvest of tagged individuals provides information on cause‐specific mortality so that both survival probability and tag‐recovery probability can be estimated from this single data type (Brownie et al., 1985; Otis & White, 2004).

With capture–recapture data, statistical power depends on both total tags deployed and the portion of those individuals reencountered after initial tagging (Williams et al., 2002). For waterfowl species in North America, it is common for <10% of tagged individuals to be recovered by hunters (Cooch et al., 2014). Thereby, it is conceivable to have apparently large data sets (tens of thousands of tagged individuals) but to still have limited data from which parameters can be estimated (Sheaffer & Malecki, 1995). Despite wide use of tag‐recovery and other capture–recapture data types, a congruent approach to quantifying and reporting the sample sizes associated with resighting, recapturing, or recovering tagged individuals is lacking.

With tag‐recovery data, total years that tagged individuals are known to be alive before being recovered (known‐fate years) is a function of total animals tagged along with both the expected lifespan and recovery probability of tagged individuals. With tag‐recovery data, direct recoveries do not contribute to known‐fate years, as these recoveries occur immediately following initial tagging when no natural mortality is assumed to occur (Brownie et al., 1985). Even though direct recoveries are required for parameter identifiability and improve the precision of parameter estimates by increasing the number of individuals for which a fate is known, these recoveries do not offer much information about the probability of survival for one or more periods in which mortality is assumed to occur (Williams et al., 2002). In the case of waterfowl, indirect recoveries are those that occur after one hunting season and one year have elapsed after being banded.

Available data in the form of total recoveries and known‐fate years (which come from indirect recoveries) affect parameter estimation when modeling tag‐recovery data, but in Bayesian models, prior distributions can also affect posterior distributions and may interact with available data to reduce the precision and accuracy of parameter estimates (Gelman et al., 2014). In the context of multivariate normal distributions, prior choice is an important consideration as recent research has shown that correlation between random effects is sensitive to the priors used (Riecke et al., 2019). In these applications, the covariance matrix of a multivariate normal distribution $\left(\sum \right)$ contains the standard deviations of random effects $\left(\sigma \right)$ and correlation between random effects $\left(\rho \right)$. Previous simulation research focused on modeling capture–recapture data with Cormack–Jolly–Seber models has shown the magnitude of correlation between random effects is underestimated when placing Wishart priors on the precision matrix $\left(\sum^{-1}\right)$ used to estimate correlated random effects (Riecke et al., 2019). These authors also report a prior formulation for the variance–covariance matrix that placed Uniform priors on the standard deviations of random effects and the correlation parameter estimated correlation with less bias than the Wishart prior these authors assessed (Riecke et al., 2019).

Correlations between demographic parameters have long been recognized as biologically meaningful (Anderson & Burnham, 1976; Nichols & Hines, 1983), and in the context of harvest management, negative correlation between the random effects of year on survival and harvest can be interpreted as evidence for harvest mortality that is additive to natural mortality (Arnold, Afton, et al., 2017). Specifically, posterior distributions of correlation between survival and recovery have been interpreted as providing evidence for strongly additive $\left(\rho <-0.7\right)$, moderately additive $\left(-0.7<\rho <-0.5\right)$, weakly additive $\left(-0.5<\rho <-0.3\right)$, or compensatory $\left(-0.3<\rho <0.3\right)$ harvest mortality (Arnold, Afton, et al., 2017). While it should be expected that the interaction between priors and effective sample size will interact along a spectrum when calculating posterior estimates of correlation between random effects (Gelman et al., 2014), guidelines like those suggested by Arnold, Afton, et al. (2017) have been recommended without assessing whether the data have enough power to robustly support these interpretations. We suggest an understanding of these interactions may be of key importance when interpreting correlation parameters for the purpose of informing harvest management.

Here, we assess the power of multivariate hierarchical models fit with Bayesian estimation to detect negative correlations between annual survival and recovery when these parameters are estimated as random effects. For our case study, we use banding and recovery data from the midcontinent mallard (*Anas platyrhychos*) population, one of the largest tag‐recovery data sets in the world, for the years 1961–1996. We focus on tag‐recovery data and the widely implemented model of Brownie et al. (1985) given its frequent application to bird and fish populations experiencing harvest or other documentable forms of anthropogenic mortality. Specifically, we evaluate model performance when choosing Wishart or Uniform priors for multivariate normal distributions (Riecke et al., 2019), along with Gamma priors like those implemented in Program MARK for multivariate normal distributions (White & Burnham, 1999). We also summarize known‐fate years for both empirical and simulated data sets while quantifying the power of our models to detect strongly negative correlation with respect to different monitoring scenarios and the three prior formulations we used to initialize Bayesian models fit to the mallard data (Johnson et al., 2015). Our case study focuses on female mallards, as we show unexpected data limitations associated with the female data when compared to the male data.

## 2 METHODS

### 2.1 Mallard banding data

We began our case study using data from mallards banded in the Central and Mississippi flyways (hereafter midcontinent mallards) as defined by the U.S. Fish and Wildlife Service [USFWS] (2017) from 1961–1996; these years were chosen because reporting probability of harvested mallards wearing metal leg bands varied little and ranged from 0.3 to 0.4 during these years (Arnold et al., 2020). The mid‐1990s also coincides with the implementation of Adaptive Harvest Management for mallards, which led to a change in the decision‐making process used to recommend harvest regulations for this population (USFWS, 2020). We acquired banding records for mallards marked with regular metal bands between 1 June and 30 September from the USGS Bird Banding Lab (BBL; Laurel, MD, USA). With the BBL banding coordinates (GISBLat, GISBLong), Program R (R Core Team, 2021) and the R package sf (Pebesma, 2018), we then filtered banding records to only include midcontinent mallards (USFWS, 2017). We included records for hatch‐year (juvenile; B_AGE_CODE = 2) and after‐hatch year (adult; B_AGE_CODE = 1, 5, 6, 8) mallards, but we excluded locals (B_AGE_CODE = 4) and individuals of unknown age class.

For these mallards, we also obtained recovery records for those individuals that were recovered by hunters (BBL code HOW = 1) between 1 September and 30 April. We excluded recoveries reported in Alaska, northern and eastern Canadian provinces (provinces NB, NL, NS, NT, NU, PE, and YT), and Mexico because recoveries from these regions were rare or band reporting probabilities are either unknown or lower than the rest of North America (Arnold et al., 2020). With these banding and recovery data, we constructed *m*‐arrays (**M**_age_) that summarize recoveries by cohort (rows) and recovery year (columns) for each age and sex class of mallards for analysis with multinomial models (Brownie et al., 1985). The *m*‐array is square for *y* years of tagging and recovery data (*Y* = 36) with total unrecovered individuals per cohort included in the *m*‐array as an additional, last column so that there are *y* rows and *y* + 1 columns. Each *m*‐array contains two recovery types: direct and indirect. Direct recoveries are found along the main diagonal of each *m*‐array and are those occurring during the hunting season beginning in the same year as tagging when no mortality is assumed to occur. Indirect recoveries are found to the right of the main diagonal of the *m*‐array (and to the left of the final column) and are those recoveries occurring at least *y* + 1 years after tagging.

To complement our empirical analysis (Table 1) and power analyses (Table 2), we report conventional summary statistics for tag‐recovery data in the form of total releases, total direct recoveries, and total indirect recoveries. In addition, we report a metric of available data in the form of total known‐fate years, average known‐fate years per year, and known‐fate years per individual tagged. We define known‐fate years as the number of years an individual was known to be alive. For example, an individual tagged in the summer of year *y* and harvested during the period of harvest beginning in year *y* + 1 would contribute one year of known‐fate data; with this approach, direct recoveries do not contribute to known‐fate year calculations.

**TABLE 1:** Summary of tag‐recovery data for midcontinent mallards captured, banded, and recovered from 1961 to 1996

	Releases	Known fate	Per year	Per release	Direct	Indirect
F HY	322,257	29,023	806	0.090	19,984	12,985
F AHY	310,295	25,787	716	0.083	10,739	11,327
M HY	375,574	80,474	2235	0.214	30,262	28,569
M AHY	584,851	127,710	3548	0.218	26,422	45,600

*Note*: Data are organized by sex, female (F) or male (M), and age at time of capture and banding, juvenile (HY), or adult (AHY). Total releases (releases), total known‐fate years (known fate), known‐fate years divided by years of study (per year), known‐fate years per individual (per individual), direct recoveries, and indirect recoveries are organized by release age and sex.

**TABLE 2:** Summary of tag‐recovery data for the 50 tag‐recovery realizations corresponding to each sampling scenario we included in our power analysis.

Cohort size	Known‐fate years	Per year	Per release	Direct	Indirect
HY_250_	902 (57)	25 (2)	0.100 (0.006)	601 (25)	406 (19)
HY_125×3;375×3_	805 (49)	22 (1)	0.089 (0.005)	546 (25)	361 (18)
HY_2,000_	6608 (145)	184 (4)	0.092 (0.002)	4363 (75)	2971 (49)
HY_10,000_	32,967 (337)	916 (9)	0.092 (0.001)	21,848 (141)	14,822 (116)
AHY_850_	2618 (89)	73 (2)	0.091 (0.003)	950 (30)	1184 (36)
AHY_550×3;1050×3_	2549 (81)	71 (2)	0.089 (0.003)	970 (25)	1149 (30)
AHY_2,000_	6546 (154)	182 (4)	0.091 (0.002)	2380 (38)	2952 (57)
AHY_10,000_	32,741 (309)	909 (9)	0.091 (0.001)	11,861 (119)	14,759 (103)

*Note*: Rows are organized by release age, juvenile (HY) or adult (AHY), while being subscripted by annual cohort size. Columns are organized to display average known‐fate years per data set (known‐fate years), average known‐fate years per year (per year), and average known‐fate years per released individual (per release), total direct recoveries (direct), and total indirect recoveries (indirect) are displayed for each monitoring scenario. The standard deviation of each summary statistic is included within parentheses.

### 2.2 Correlation models

**FIGURE 1:**
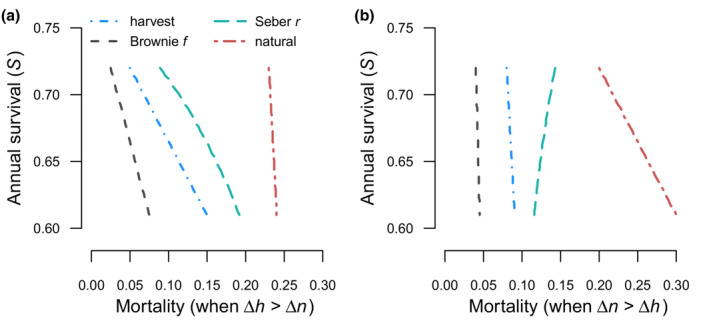
Two scenarios of additive harvest demonstrating different behavior of the Seber *r* recovery formulation relative to the Brownie *f* formulation. Each figure displays the relationship between survival probability (*S*) and four metrics of annual mortality: annual harvest probability (*h*; dot‐dash line in blue), Brownie *f* recovery probability when reporting probability is 0.5 (*f*; dashed line in gray), Seber *r* recovery parameter (*r*; long‐dash line in green), annual natural mortality (*n*; dash‐short‐dash line in red). The Brownie recovery probabilities are the product of harvest and reporting probability (*f* = *h ×* 0.5). The Seber recovery values are a function of survival and recovery (*r* = *f*/(1 – *S*)). Natural mortality is the difference between 1, survival, and harvest (*n* = 1 – *S* – *h*). In panel 1a (Δ*h* > Δ*n*), both the Brownie or Seber model parameterizations indicate a negative relationship between survival and harvest in Figure 1a. In Figure 1b (Δ*n* > Δ*h*), the Brownie parameterization indicates a negative relationship, while the Seber parameterization indicates a positive relationship between survival and harvest (which is the opposite of additive harvest).

We parameterized the multinomial formulation of the Brownie et al. (1985) band‐recovery model in a Bayesian framework to estimate annual survival $\left(S\right)$ and recovery $\left(f\right)$ probabilities for two age classes (Brownie et al., 1985, Kéry & Schaub, 2012 pp. 248–255), juvenile (i.e., hatch year; HY) and adult (i.e., after‐hatch year; AHY). A common notation for this model structure would be *S*_*age,year*_ *f*_*age,year*_; we exclude “sex” from the notation as we analyzed the female and male data separately. With this model formulation, the recovery parameter (*f*) is the joint probability of being shot, retrieved, and reported (Brownie et al., 1985). We note the models specified by Kéry and Schaub (pp. 248–255) implement the Seber *r* parameterization in place of the Brownie *f* parameterization, which is a difference regarding the estimation of recovery probability (Cooch et al., 2014 pp. 246–248). When specifying the Brownie *f* parameterization, we did not distinguish between direct recovery probability and indirect recovery probability. We did not parameterize models with the Seber *r* formulation (Sedinger et al., 2010) as this method is prone to providing incorrect inference when temporal variation in natural mortality exceeds variation in harvest mortality (Figure 1; Code available in supporting information online). While future work could further assess the Seber *r* parameterization for correlation analyses, the mathematical difficulties demonstrated in Figure 1 preclude us from further considering this parametrization here.

We estimated the probability of observed tag‐recovery data $\left(M_{\mathrm{age},y,1:Y}\right)$ using multinomial distributions with success probabilities for juveniles $\left(\alpha \right)$ and adults $\left(\beta \right)$ and the total individuals released (**R**_*age*_) during each year (Equation 1).

(1) $$\begin{array}{l} M_{\mathrm{HY},y,1:\left(Y+1\right)}∼\text{Multinomial}\left(\alpha_{y,1:Y},R_{\mathrm{HY},y}\right)\;\\ M_{\mathrm{AHY},y,1:\left(Y+1\right)}∼\text{Multinomial}\left(\beta_{y,1:Y},R_{\mathrm{AHY},y}\right) \end{array}$$

We defined the age‐specific cell probabilities as a function of annual survival and recovery probability. Cell‐probabilities corresponding to direct recoveries (along the main diagonal) are a function of annual recovery probabilities while cell probabilities corresponding to indirect recoveries (off the main diagonal) are a function of annual survival and recovery probabilities. Age‐specific cell probabilities corresponding to release year *i* (rows) and recovery year *j* (columns) are displayed in Equation 2. Note, the *m*‐arrays do not contain data below and left of the main diagonal $\left(i>j\right)$.
(2)

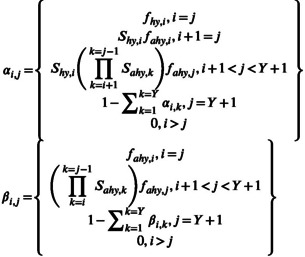




We estimated annual survival and recovery probabilities on the logit scale while using random effects to model annual variation in survival $\left(\varepsilon_{S_{y}}\right)$ and recovery $\left(\varepsilon_{f_{y}}\right)$ relative to the hierarchical mean survival $\left(\mu_{S}\right)$ and recovery $\left(\mu_{f}\right)$ probability for each age class (Equations 3, 4). Neither the hierarchal means nor random effects were shared between age classes, thereby we omit the age notation from our equations for clarity. During initial modeling, our models occasionally failed during the initial period of estimation; we avoided this problem by choosing vague priors for mean survival (Equation 3) and mean recovery (Equation 4) on the natural scale while restricting these priors to a narrower range than the possible parameter space of 0 and 1. These priors are uninformative relative to the range of biologically plausible values of survival and recovery experienced by mallards.

(3) $$\begin{array}{l} \log\mathrm{it}\left(S_{y}\right)=\mu_{S}+\varepsilon_{s_{y}}\\ \mu_{S}=\log\left({\text{mean}}_{S}/\left(1-{\text{mean}}_{S}\right)\right)\\ {\text{mean}}_{S}\sim \text{Uniform}\left(0.10,0.99\right)\; \end{array}$$

(4) $$\begin{array}{l} \log\mathrm{it}\left(f_{y}\right)=\mu_{f}+\varepsilon_{f_{y}}\\ \mu_{f}=\log\left({\text{mean}}_{f}/\left(1-{\text{mean}}_{f}\right)\right)\\ {\text{mean}}_{f}\sim \text{Uniform}\left(0.01,0.30\right)\; \end{array}$$

We drew random effects from multivariate normal distributions with a mean of 0 and a variance–covariance matrix $\left(\sum \right)$ or precision matrix $\left(\sum^{-1}\right)$ depending on which of the three priors we specified for these matrices. Our decision to place priors on the variance–covariance matrix or the precision matrix was due to practical limitations in Program JAGS related to ensuring these matrices are positive definite (Plummer, 2003). Here, we consider three models that differ in the priors we used to initiate multivariate normal distributions.

The first prior was a Wishart distribution (Link & Barker, 2005; Riecke et al., 2019), which is a conjugate prior for the precision matrix $\left(\sum^{-1}\right)$ of a multivariate normal distribution (Equation 5). With this Wishart prior, random effects were drawn from multivariate normal distributions with a mean of 0 and a precision matrix (alternatively, hierarchical mean survival and recovery could be used as the mean values for these normal distributions instead of 0).

(5) $$\left[\begin{array}{l} \varepsilon_{S_{y}}\\ \varepsilon_{f_{y}} \end{array}\right]\sim \text{Normal}\left(\left[\begin{array}{l} 0\\ 0 \end{array}\right],\sum^{-1}\right)$$

$$\sum^{-1}\sim \text{Wishart}\left(3,I\right)$$

$$I=\left(\begin{array}{cc} 1 & 0\\ 0 & 1 \end{array}\right)$$

Our second prior formulation was for individual parameters within the covariance matrices. Specifically, we used a Uniform prior for the standard deviations of random effects for survival and recovery $\left(\sigma_{s}\;\text{and}\;\sigma_{f},,\text{respectively}\right)$ and a vague prior for correlation between survival and recovery $\left(\rho \right)$ as specified in Equation 6 (Riecke et al., 2019). Our prior for correlation is vague like a single Uniform distribution prior, but the use of a Beta distribution would allow for a shaped prior if desired. When implementing these flat priors, random effects for survival and recovery were drawn from multivariate normal distributions with a mean of 0 and variance–covariance matrix using the JAGS function dmnorm.vcov (Plummer, 2003).

(6) $$\left[\begin{array}{c} \varepsilon_{S_{y}}\\ \varepsilon_{f_{y}} \end{array}\right]\sim \text{Normal}\left(\left[\begin{array}{c} 0\\ 0 \end{array}\right],\sum \right)$$

$$\sum =\left(\begin{array}{cc} \sigma_{S}^{2} & \sigma_{S}\sigma_{f}\rho \\ \sigma_{S}\sigma_{f}\rho & \sigma_{f}^{2} \end{array}\right)$$

$$\sigma_{S}\sim \text{Uniform}\left(0,5\right)$$

$$\sigma_{f}\sim \text{Uniform}\left(0,5\right)$$

$$\rho \sim \left(2\times \rho_{\text{prior}}\right)-1$$

$$\rho_{\text{prior}}∼\text{Beta}\left(1,1\right)$$

Our third prior formulation placed priors on components of the precision matrix which is like the default priors specified for multivariate normal distributions in Program MARK (White & Burnham, 1999). In this case, we specified the distribution Gamma(1.001,0.001) for the parameters found along the main diagonal of the precision matrix $\left(1/\sigma^{2}\right)$ and a flat prior for the equivalent of a correlation parameter $\left(\rho^{*}\right)$ for the precision matrix (Equation 7). With this prior, random effects were estimated from multivariate normal distributions with mean values of 0 and a precision matrix. Here, we calculated correlation from the variance–covariance matrix after inverting the precision matrix in Program JAGS (Plummer, 2003).

(7) $$\left[\begin{array}{l} \varepsilon_{S_{y}}\\ \varepsilon_{f_{y}} \end{array}\right]\sim \text{Normal}\left(\left[\begin{array}{l} 0\\ 0 \end{array}\right],\sum^{-1}\right)$$

$$\sum^{-1}=\left(\begin{array}{cc} 1/\sigma_{S}^{2} & 1/\sigma_{S}\left(1/\sigma_{f}\right)\rho^{*}\\ 1/\sigma_{S}\left(1/\sigma_{f}\right)\rho^{*} & 1/\sigma_{f}^{2} \end{array}\right)$$

$$1/\sigma_{S}^{2}\sim \text{Gamma}\left(1.001,0.001\right)$$

$$1/\sigma_{f}^{2}\sim \text{Gamma}\left(1.001,0.001\right)$$

$$\rho^{*}\sim \left(2\times \rho_{\text{prior}}^{*}\right)-1$$

$$\rho_{\text{prior}}^{*}∼\text{Beta}\left(1,1\right)$$

Hereon, we refer to these three prior formulations as “Wishart” (Equation 5), “Uniform” (Equation 6), or “Gamma” (Equation 7) priors. For clarity, we consistently present results in order of juvenile females, adult females, juvenile males, and adult males.

We fit models in JAGS Version 4.3.0 (Plummer, 2003) in Program R using the R packages coda (Plummer et al., 2006) and jagsUI (Kellner, 2019) by running four MCMC chains for 50,000 iterations while applying a thin rate of 10 after discarding the first 10,000 values of each chain, leaving us with a posterior sample of 16,000 values for each parameter. We assessed convergence by inspecting trace plots for consistent chain mixing while also reviewing $\hat{R}$ values (Gelman et al., 2014).

### 2.3 Power analysis

**FIGURE 2:**
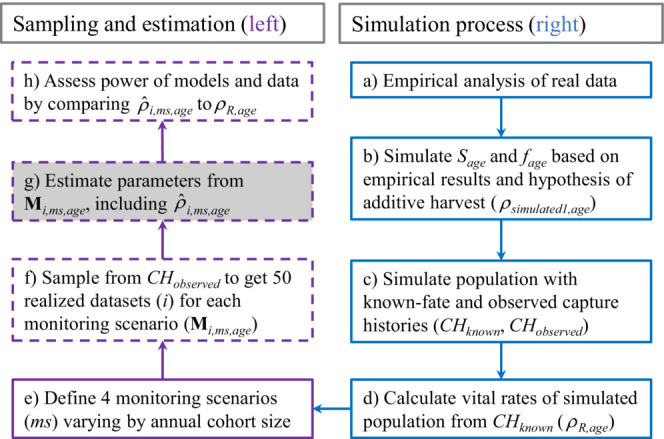
Schematic depicting the steps of our power analysis. Beginning in the upper right, simulation steps are displayed (blue borders) starting at the top and moving clockwise. Beginning at lower left, sampling and estimation steps are shown (purple borders). Boxes with solid borders correspond to steps completed once while boxes with dashed lines correspond to steps were repeated 200 times (4 scenarios × 50 data realizations). The box focusing on parameter estimation is shaded as it is usually the step of greatest interest in both empirical and simulation studies.

After fitting our models to the mallard data, we assessed our power to detect negative correlation between survival and recovery with the models we used for the empirical analysis (Figure 2); our approach was guided by a simple question, “will my study answer my research question?” (Johnson et al., 2015). We began by simulating age‐specific survival and recovery probabilities for a 36‐year period using multivariate normal distributions and parameters we estimated for female mallards with Uniform priors while also specifying correlation between survival and recovery to be −0.8 for each age class (Figure 2a). Our simulation of annual survival and recovery probabilities used the following parameters we estimated for juvenile female mallards; juvenile mean survival $\left({\hat{\mu }}_{S_{\mathrm{HY}}}=0.574\right)$ and recovery $\left({\hat{\mu }}_{f_{\mathrm{HY}}}=0.061\right)$, along with the standard deviations of the random effects for juvenile survival $\left({\hat{\sigma }}_{S_{\mathrm{HY}}}=0.184\right)$ and recovery $\left({\hat{\sigma }}_{f_{\mathrm{HY}}}=0.269\right)$. For adults, we used mean survival $\left({\hat{\mu }}_{S_{\mathrm{AHY}}}=0.571\right)$ and recovery $\left({\hat{\mu }}_{f_{\mathrm{AHY}}}=0.032\right)$ and the standard deviations of the random effects estimated for survival $\left({\hat{\sigma }}_{S_{\mathrm{AHY}}}=0.131\right)$ and recovery $\left({\hat{\sigma }}_{f_{\mathrm{AHY}}}=0.312\right)$. For each age class, these simulated probabilities were consistent with additive harvest mortality when quantified using two metrics (Figure 2b): (1) correlation was strongly negative $\left(\rho_{\text{simulated}1,\mathrm{HY}}=-0.801,,\rho_{\text{simulated}1,\mathrm{AHY}}=-0.792\right)$ and (2) the estimated relationship between survival and recovery $\left(\beta \right)$ was about −2.5 $\left(\beta_{\text{simulated}1,\mathrm{HY}}=-2.420,,\beta_{\text{simulated}1,\mathrm{AHY}}=-2.408\right)$. The condition β=−2.5 is consistent with the hypothesis of additive harvest mortality when band‐reporting probability is 0.4 (Appendix S1), which is close to the estimated band‐reporting probability during the years of our study (Arnold et al., 2020).

**FIGURE 3:**
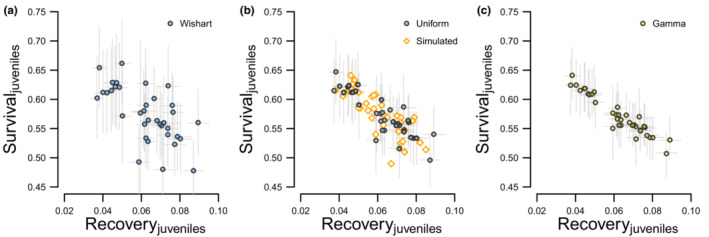
First‐winter survival and recovery estimated for female midcontinent mallards banded as juveniles from 1961 to 1996. Each figure is for the same data such that the difference between Figures 3a–c is a result of the prior used to initiate multivariate normal distributions; Wishart (Figure 3a), Uniform (Figure 3b), and Gamma (Figure 3c). The real parameters we simulated for our power analysis are displayed in Figure 3b (orange diamonds).

**FIGURE 4:**
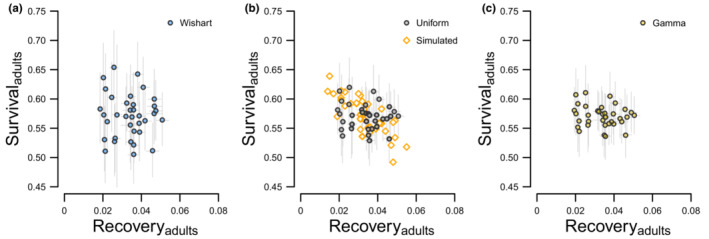
Estimates of adult survival and recovery estimated for female midcontinent mallards banded from 1961–1996. Each figure is for the same data such that the difference between Figures 4a–c is a result of the prior used to initiate multivariate normal distributions; Wishart (Figure 4a), Uniform (Figure 4b), and Gamma (Figure 4c). The real parameters we simulated for our power analysis are displayed in Figure 4b (orange diamonds).

With the annual survival and recovery probabilities that we simulated for each class (Figure 3b, 4b), we simulated known‐fate histories for an entire population of individuals (*CH*_*known*_). From these known‐fate capture histories, we simulated observed capture histories (*CH*_*observed*_) only containing initial encounters and recoveries (Figure 2c). Because we developed our power analysis to emphasize the importance of sampling (see below), *CH*_*known*_ and *CH*_*observed*_ were composed of histories for 9 million individuals initially encountered in the juvenile or hatch‐year age class (250,000 new individuals per year) and 14.4 million individuals initially encountered in the adult or after‐hatch year age class (400,000 new individuals per year) so that each simulated individual had a known fate and observed capture history. After simulating *CH*_*known*_ and *CH*_*observed*_, we calculated the realized annual survival and recovery probabilities for both age classes from the known‐fate capture histories (Figure 2d), as well as realized correlation between age‐specific survival and recovery probabilities (*ρ*_*R*,age_). We used the realized correlation values for each age class (*ρ*_*R*,HY_ = −0.801, *ρ*_*R,AHY*_ = −0.787) as reference values for true correlation of our simulated population in subsequent steps of our power analysis.

After all simulations were complete (Figure 2a–d), we then defined four monitoring scenarios that differed in how many individuals were sampled and tagged during each year of our simulation study (Figure 2e). These four monitoring scenarios were (1) modest and constant (HY_250_, AHY_800_); (2) modest and episodic in 3‐year cycles (HY_125 × 3;375 × 3_, AHY_550 × 3;1050 × 3_); (3) intermediate (HY_2,000_, AHY_2,000_); and (4) intensive (HY_10,000_, AHY_10,000_). The modest scenarios are comparable to sample sizes available for female lesser scaup (*Aythya affinis*; Arnold et al., 2016), the intermediate scenario is like sample sizes available for northern pintails (*Anas acuta*; Bartzen & Dufour, 2017), and the intensive scenario is more like the sample sizes available for mallards. We then randomly sampled capture histories from *CH_observed_* using the criteria of each monitoring scenario to obtain 200 realized data sets, 50 for each monitoring scenario (Figure 2f). For data set *i* from monitoring scenario *ms*, we summarized the sampled capture histories to *m*‐array format (**M**_*i,ms,age*_). We sampled without replacement within each data set (**M**_*i,ms,age*_) and with replacement among data sets **M**_*i,ms,age*_.

We then fit the same three Brownie models to each data set **M**_*i,ms,age*_ (with Wishart, Gamma, and Uniform priors) to estimate the same parameters as for the mallard data (Figure 2g), including correlation between survival and recovery $\left({\hat{\rho }}_{i,\mathrm{ms},\mathrm{age}}\right)$. We evaluated our power to detect strongly negative correlation by comparing median (50th quantile) estimates of ${\hat{\rho }}_{i,\mathrm{ms},\mathrm{age}}$ to *ρ*_*R,age*_. We also calculated the portion of the posterior estimates of correlation that fell within the bins that associate values of correlation to varying degrees of additive mortality (Figure 2h) suggested by Arnold, Afton, et al. (2017). Our power analysis focused on the combination of sampling and parameter estimation to capture truth of a population, which in our case is *ρ*_*R,age*_. We did not focus on the ability of a model to estimate parameters from a sample (Riecke et al., 2019). Both approaches have merits depending on intention, and our intention was to focus on our ability to detect strongly negative correlation; this goal is best served by the approach we implemented here.

## 3 RESULTS

### 3.1 Mallard analysis

Based on age at banding, we obtained records for 322,257 hatch‐year females, 310,295 after‐hatch year females, 375,574 individuals as hatch‐year males, and 584,851 after‐hatch year males (Table 1). On a per individual basis, there were twice as many known‐fate years for every male (≈0.2) as there was for every female (≈0.1), but expected known‐fate years per individual did not meaningfully vary within sex by release age (Table 1).

**FIGURE 5:**
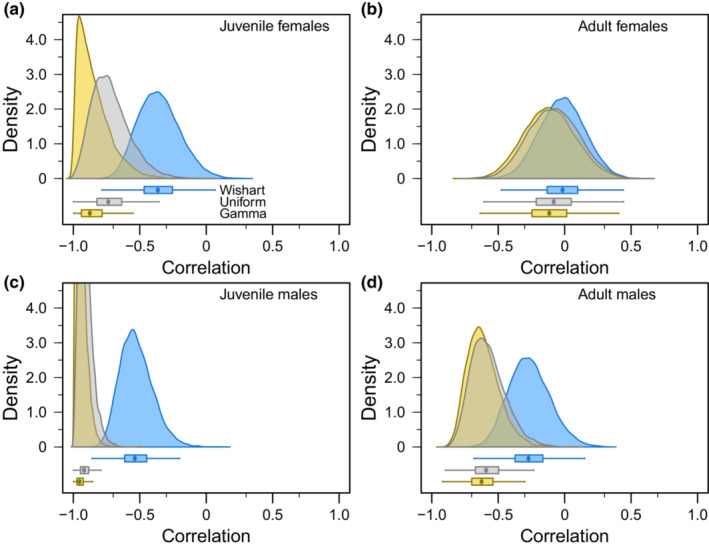
Posterior distributions of correlation between survival and recovery estimated for juvenile female (Figure 5a), adult female (Figure 5b), juvenile male (Figure 5c), and adult male (Figure 5d) midcontinent mallards banded from 1961 to 1996. Posterior distributions are displayed as density plots and horizontal boxplots while being color coded by prior; Wishart (blue), Uniform (gray), and Gamma (yellow). From left to right, the minor x‐axis ticks represent −0.7, −0.3, 0.3, and 0.7. The maximum density values for the Uniform and Gamma posteriors in Figure 5c are ≈9.2 and ≈13.4, respectively.

All estimated and derived parameters converged ($\hat{R}$ < 1.1), and importantly, posterior distributions of survival and recovery (Figures 3, 4) as well as posterior distributions of correlation between random effects were updated by the data such that the estimates did not span the parameter space (Figure 5). Survival was generally estimated to be between 0.5 and 0.7, which is near the middle of the logit parameter space, while recovery was generally <0.1, which is near the lower bound of the logit parameter space (Table 3).

**TABLE 3:** Survival ($\hat{S}$) and recovery ($\hat{f}$) probabilities we estimated for midcontinent mallards between 1961 and 1996.

	${\hat{\mu }}_{s}\left(\mathrm{BSD}\right)$	${\hat{S}}_{\min}\left(\mathrm{BSD}\right)$	${\hat{S}}_{\max}\left(\mathrm{BSD}\right)$	${\hat{\mu }}_{f}\left(\mathrm{BSD}\right)$	${\hat{f}}_{\min}\left(\mathrm{BSD}\right)$	${\hat{f}}_{\max}\left(\mathrm{BSD}\right)$
W_F,HY_	0.576 (0.013)	0.478 (0.025)	0.662 (0.036)	0.061 (0.003)	0.037 (0.002)	0.089 (0.003)
U_F,HY_	0.574 (0.010)	0.496 (0.022)	0.647 (0.028)	0.061 (0.003)	0.037 (0.002)	0.089 (0.003)
G_F,HY_	0.573 (0.009)	0.509 (0.020)	0.640 (0.023)	0.061 (0.002)	0.037 (0.002)	0.089 (0.003)
W_F,AHY_	0.572 (0.011)	0.505 (0.026)	0.654 (0.031)	0.032 (0.002)	0.019 (0.001)	0.051 (0.002)
U_F,AHY_	0.571 (0.006)	0.529 (0.021)	0.620 (0.024)	0.032 (0.002)	0.019 (0.001)	0.051 (0.002)
G_F,AHY_	0.571 (0.005)	0.536 (0.019)	0.610 (0.021)	0.033 (0.002)	0.019 (0.001)	0.051 (0.002)
W_M,HY_	0.604 (0.013)	0.498 (0.018)	0.725 (0.024)	0.079 (0.003)	0.054 (0.002)	0.111 (0.003)
U_M,HY_	0.603 (0.011)	0.500 (0.016)	0.711 (0.019)	0.080 (0.003)	0.054 (0.002)	0.111 (0.003)
G_M,HY_	0.602 (0.011)	0.502 (0.015)	0.706 (0.017)	0.080 (0.003)	0.054 (0.002)	0.111 (0.003)
W_M,AHY_	0.676 (0.009)	0.615 (0.016)	0.739 (0.015)	0.046 (0.002)	0.029 (0.001)	0.063 (0.002)
U_M,AHY_	0.676 (0.006)	0.617 (0.013)	0.730 (0.014)	0.045 (0.002)	0.029 (0.001)	0.064 (0.002)
G_M,AHY_	0.676 (0.005)	0.618 (0.013)	0.730 (0.014)	0.045 (0.002)	0.029 (0.001)	0.064 (0.001)

*Note*: Results are for juvenile (HY) and adult mallards (AHY) of both sexes, female (F) and (M), and are organized by prior distribution (Wishart: W; Gamma: G; Uniform: U). For both survival and recovery, respectively, we report the hierarchal mean of survival and recovery $\left({\hat{\mu }}_{S},,,{\hat{\mu }}_{f}\right)$, the minimum annual estimate $\left({\hat{S}}_{\min},,,{\hat{f}}_{\min}\right)$, and the maximum annual estimate $\left({\hat{S}}_{\max},,,{\hat{f}}_{\max}\right)$. The Bayesian standard deviation (BSD) of each parameter is displayed after the point estimates.

If correlation between the annual random effects for survival and recovery is interpreted using previously suggested guidelines (Arnold, Afton, et al., 2017; Arnold, Clark, et al., 2017), our interpretation of additive mortality depended on prior choice (Figure 5). With Wishart priors, harvest mortality would be interpreted as weakly additive for juvenile females $\left(\hat{\rho }=-0.364\;\left[-0.632,-0.026\right]\right)$, compensatory for adult females $\left(\hat{\rho }=-0.016\;\left[-0.340,0.314\right]\right)$, and moderately additive for juvenile males $\left(\hat{\rho }=-0.537\;\left[-0.733,-0.253\right]\right)$, but weakly additive or compensatory for adult males $\left(\hat{\rho }=-0.272\;\left[-0.548,0.061\right]\right)$. Using Uniform priors and the same data, harvest mortality appears moderately to strongly additive for juvenile females $\left(\hat{\rho }=-0.737\;\left[-0.940,-0.388\right]\right)$, compensatory for adult females $\left(\hat{\rho }=-0.082\;\left[-0.437,0.294\right]\right)$, strongly additive for juvenile males $\left(\hat{\rho }=-0.919\;\left[-0.982,-0.784\right]\right)$, and moderately additive for adult males $\left(\hat{\rho }=-0.591\;\left[-0.789,‐0.274\right]\right)$. Gamma priors provided results generally similar to Uniform priors (Figure 5) with harvest mortality appearing strongly additive for juvenile females $\left(\hat{\rho }=-0.876\;\left[-0.987,-0.539\right]\right)$, compensatory for adult females $\left(\hat{\rho }=-0.116\;\left[-0.473,0.271\right]\right)$, strongly additive for juvenile males $\left(\hat{\rho }=-0.953\;\left[-0.991,-0.846\right]\right)$, and moderately additive for adult males $\left(\hat{\rho }=-0.625\;\left[-0.807,-0.337\right]\right)$.

**FIGURE 6:**
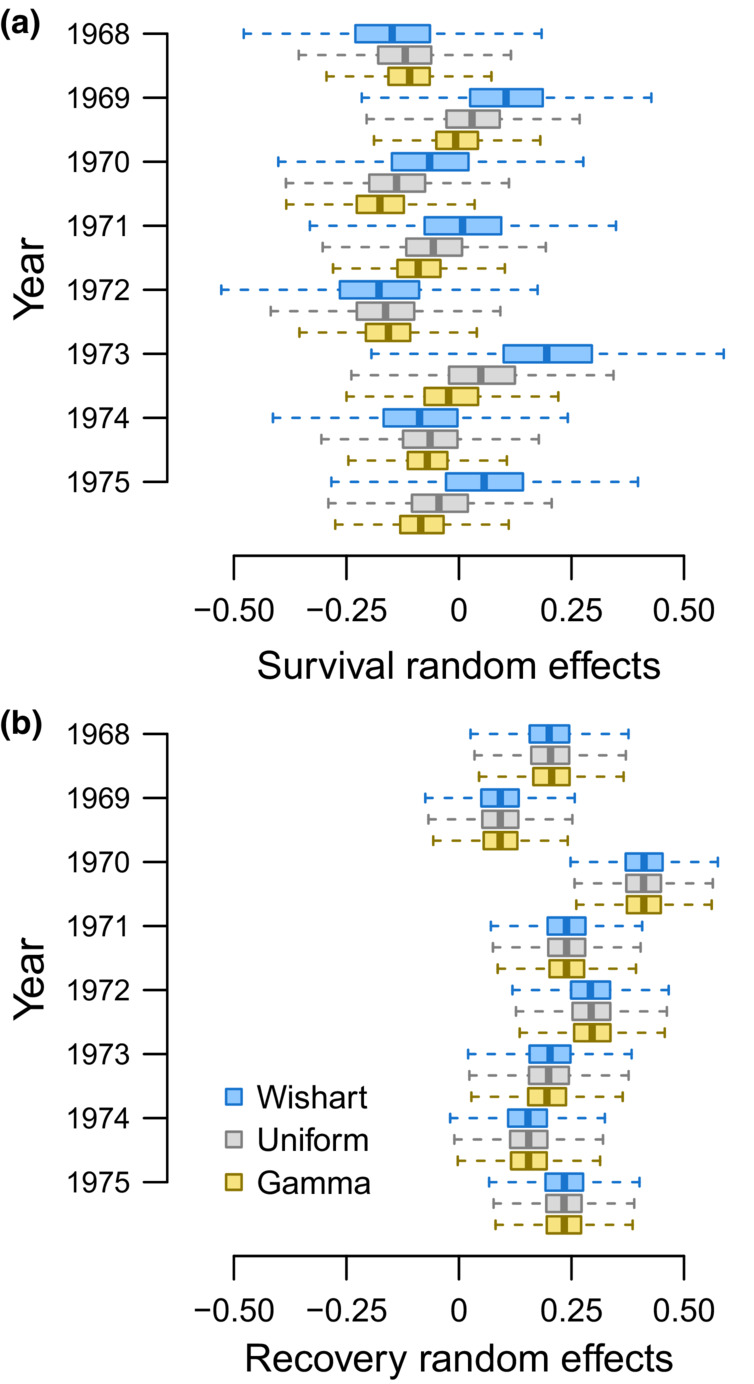
Random effect estimates of first‐winter survival (Figure 6a) and recovery (Figure 6b) for juvenile female midcontinent mallards banded from 1968 to 1975. Estimates differ by the prior distribution used to initiate multivariate normal distributions; Wishart (blue), Uniform (gray), and Gamma (yellow) priors. Note these values are on the logit scale and were estimated relative to their hierarchal means.

Inference about the variability of mean and annual mallard survival also depended on prior choice (Table 3). Annual survival estimates were most variable when we fit models using Wishart priors while models fit using Uniform priors were slightly more variable than models fit using Gamma priors (Figures 3, 4, 6). This variability was evident by (1) greater Bayesian standard deviations for mean survival estimates (Table 3 column 1) and (2) greater differences between the minimum and maximum annual survival estimates relative to the estimated hierarchal means (Table 3 columns 2–3); and (3) less precisely estimated random effects (Figure 6). Unlike survival, the variability of mean recovery and annual recovery estimates was generally insensitive to prior choice (Figure 6, Table 3 columns 4–6).

### 3.2 Power analysis

**FIGURE 7:**
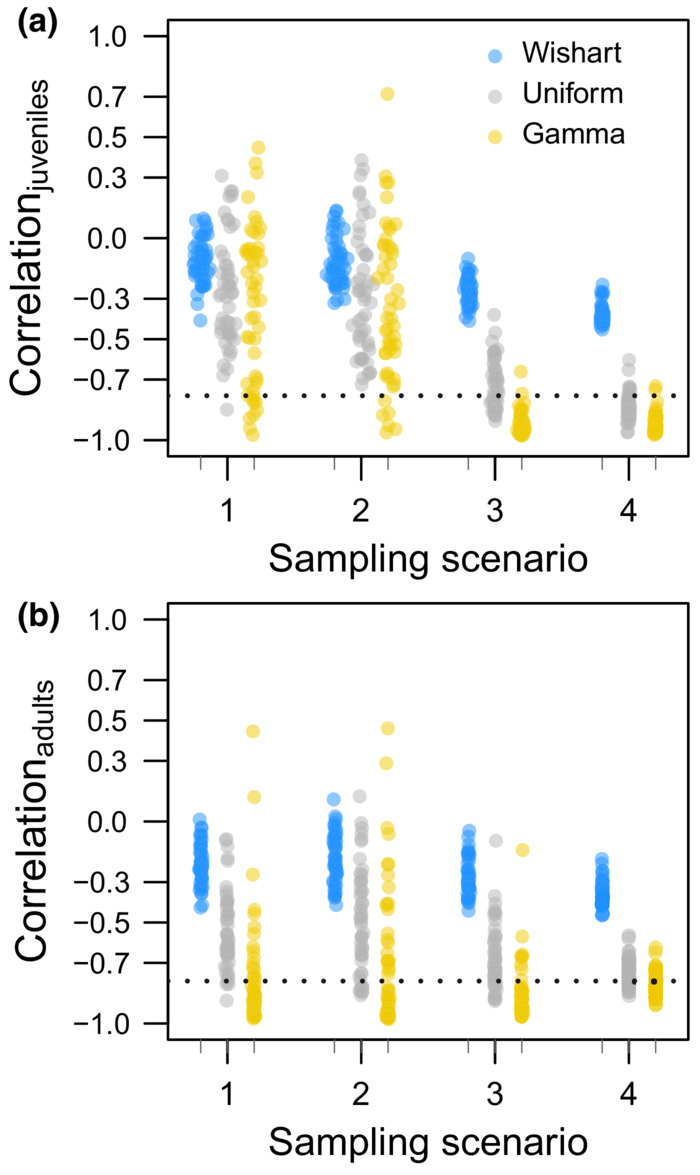
Median estimates of correlation displayed by each prior distribution and monitoring scenario combination; Wishart (blue), Uniform (gray), and Gamma (yellow). Fifty estimates are displayed for each combination of age, monitoring scenario, and prior distribution. Points are horizontally jittered by the difference between the hierarchical mean survival estimate $\left(\mu_{S}\right)$ for each age class and the age‐specific median survival probabilities of our simulated population (*S*_HY_ = 0.576 and *S*_AHY_ = 0.572). For reference, the mean survival estimates for juveniles in sampling scenario 1 estimated with Wishart priors ranges from 0.525 to 0.646, and these points are jittered relative to the value of 0.576. The horizontal lines (dashed) correspond to true correlation between survival and recovery (*ρ*_*R,*HY_ = −0.801, *ρ*_*R,*AHY_ = −0.787).

**FIGURE 8:**
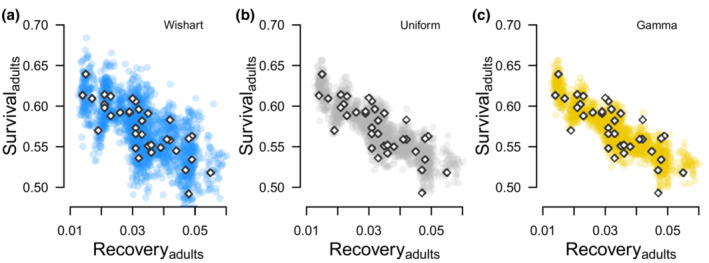
Median values of annual survival and recovery of adults estimated from 50 data realizations corresponding to our intensive monitoring (10,000 tags per age class deployed annually). In each figure, annual estimates of adult survival and recovery (filled circles) are displayed by prior distribution such that there are 1800 points (36 years × 50 data realizations) in each plot. True adult survival and recovery experienced by adults in our simulated population are shown as diamonds in each figure.

Our power analysis indicated data from intensive monitoring and models fit with Uniform priors recovered our reference values of correlation between survival and recovery $\left(\rho_{R,\mathrm{HY}}=-0.801,\rho_{R,\mathrm{AHY}}=-0.787\right)$ with more reliability than any other combination of monitoring scenario and prior choice (Table 4, Appendix S2). With modest data, the opposite was true as we had little, if any, power to detect negative correlation between survival and recovery (Table 4, Appendix S2). Correlation was more likely to be estimated with severe bias and higher sensitivity to both effective sample size and prior choice than mean survival (Figure 7). Prior influence extended beyond estimates of correlation to estimates of annual survival, but not annual recovery, with annual survival being most variable when estimated using Wishart priors and less similarly less variable when estimated with Uniform or Gamma priors (Figure 8). Below, we summarize our results by the intensive, intermediate, and modest monitoring scenarios.

**TABLE 4:** Summary of age‐specific correlation estimated for each monitoring scenario and prior distribution (Wishart, Uniform, Gamma) that we considered in our power analysis

	${\hat{\rho }}_{\text{median}}$	${\hat{\rho }}_{\left[<-0.7\right]}$	${\hat{\rho }}_{\left[>-0.7,<-0.5\right]}$	${\hat{\rho }}_{\left[>-0.5,<-0.3\right]}$	${\hat{\rho }}_{\left[>-0.3,<0.3\right]}$	${\hat{\rho }}_{\left[>0.3\right]}$
Hatch year						
Wishart_250_	−0.114	0.004	0.061	0.184	0.681	0.070
Uniform_250_	−0.304	0.225	0.145	0.133	0.298	0.200
Gamma_250_	−0.342	0.348	0.091	0.076	0.201	0.284
Wishart_125 × 3;375 × 3_	−0.121	0.006	0.072	0.191	0.652	0.080
Uniform_125 × 3;375 × 3_	−0.319	0.238	0.145	0.129	0.292	0.197
Gamma_125 × 3;375 × 3_	−0.364	0.362	0.089	0.071	0.185	0.294
Wishart_2,000_	−0.268	0.003	0.096	0.337	0.561	0.004
Uniform_2,000_	−0.745	0.568	0.220	0.117	0.084	0.011
Gamma_2,000_	−0.937	0.865	0.068	0.029	0.028	0.009
Wishart_10,000_	−0.377	0.006	0.200	0.473	0.320	0
Uniform_10,000_	−0.841	0.834	0.139	0.023	0.004	0
Gamma_10,000_	−0.928	0.947	0.046	0.006	0.001	0
After‐hatch year						
Wishart_250_	−0.217	0.004	0.092	0.267	0.616	0.021
Uniform_250_	−0.577	0.365	0.210	0.155	0.213	0.056
Gamma_250_	−0.865	0.659	0.087	0.052	0.099	0.102
Wishart_550 × 3;1050 × 3_	−0.187	0.004	0.083	0.240	0.639	0.034
Uniform_550 × 3;1050 × 3_	−0.546	0.359	0.179	0.138	0.235	0.089
Gamma_550 × 3;1050 × 3_	−0.845	0.616	0.084	0.054	0.113	0.132
Wishart_2,000_	−0.283	0.005	0.120	0.342	0.528	0.005
Uniform_2,000_	−0.708	0.512	0.244	0.129	0.106	0.008
Gamma_2,000_	−0.891	0.797	0.108	0.046	0.042	0.007
Wishart_10,000_	−0.352	0.005	0.168	0.444	0.383	0
Uniform_10,000_	−0.753	0.643	0.283	0.063	0.011	0
Gamma_10,000_	−0.812	0.795	0.174	0.027	0.004	0

*Note*: For each monitoring scenario (designated by cohort size in subscripts), the results for each prior correspond to the same 50 data sets. The median correlation estimate from each monitoring and prior combination is displayed first followed by the portion of these estimates that fell within the subscripted values. True correlation was $\rho_{R,\mathrm{HY}}=-0.801$ (juveniles) and $\rho_{R,\mathrm{AHY}}=-0.787$ (adults). Figures of these posterior distributions are displayed in Appendix S2.

#### 3.2.1 Intensive monitoring scenario

The median estimate of correlation from all data realizations corresponding to our intensive monitoring scenario (10,000 tags per age class annually) was close to our reference values for correlation 

$$\left(\rho_{R,HY}=‐0.801,\rho_{R,AHY}=‐0.787\right)$$

when we used Uniform priors $\left({\hat{\rho }}_{\mathrm{HY}}=-0.841,{\hat{\rho }}_{\mathrm{AHY}}=-0.753\right)$. With Gamma priors, correlation estimates were more negative than when we fit models using Uniform priors $\left({\hat{\rho }}_{\mathrm{HY}}=-0.928,{\hat{\rho }}_{\mathrm{AHY}}=-0.812\right)$ with results for juveniles being more sensitive and more negative than results for adults (Table 4). With intensive monitoring and Uniform priors, about 83% and 64% of the posterior estimates of correlation were in the range $\hat{\rho }<-0.7$ for juveniles and adults, respectively. Implementing Gamma priors resulted in about 93% and 81% of posterior estimates of correlation for juveniles and adults, respectively, falling in the range $\hat{\rho }<-0.7$. For these same 50 data realizations, median estimates of correlation substantially underestimated negative correlation relative to our reference values when we fit models with Wishart priors $\left({\hat{\rho }}_{\mathrm{HY}}=-0.377,{\hat{\rho }}_{\mathrm{AHY}}=-0.352\right)$. With Wishart priors and intensive monitoring, the interpretation of weakly additive harvest was most supported for juveniles and adults as 47% and 44% of the posterior estimates of correlation fell in the range $-0.5<\hat{\rho }<-0.3$, respectively (Table 4). At the same time, the interpretation of compensatory harvest received similar levels of support as 32% of the correlation estimates for juveniles and 38% of the correlation estimates for adults fell in the range $-0.3<\hat{\rho }<0.3$. The posterior estimates of correlation obtained from intensive data with Wishart priors are peaked and do not span the parameter space of −1 and 1 despite substantially underestimating correlation relative to our reference values (Appendix S2). With the intensive data realizations, we also evaluated five formulations of the Gamma prior ranging from Gamma(1.1,0.1) to Gamma(1.00001,0.00001); these 5 priors had remarkably variable influence on posterior inference (Appendix S3).

#### 3.2.2 Intermediate monitoring scenario

When cohort size was intermediate for both age classes (2000 tags per age class annually), the median correlation estimate from all 50 data realizations was modestly underestimated using Uniform priors $\left({\hat{\rho }}_{\mathrm{HY}}=-0.708,{\hat{\rho }}_{\mathrm{AHY}}=-0.745\right)$ when compared to our reference values of $\rho_{R,\mathrm{HY}}=-0.801$ and $\rho_{R,\mathrm{AHY}}=-0.787$. The portion of these correlation estimates falling in the range $\hat{\rho }<-0.7$ was 57% for juveniles and 51% for adults, thereby providing less support for the interpretation of strongly additive harvest when compared to the intensive monitoring scenario. Models fit with Gamma priors to the same 50 data realizations provided more negative median correlation estimates than Uniform priors $\left({\hat{\rho }}_{\mathrm{HY}}=-0.937,{\hat{\rho }}_{\mathrm{AHY}}=-0.891\right)$ and were more negative when compared to results from the combinations of intensive monitoring and Gamma priors or intermediate monitoring and Uniform priors (Table 4, Appendix S2). Of the 12 monitoring and prior combinations we considered, it was this combination of Gamma priors and intermediate monitoring that produced the most negative values as 87% of the correlation estimates for juveniles and 80% of the correlation estimates for adults fell in the range $\hat{\rho }<-0.7$ (Table 4). For the same intermediate data in which correlation between survival and recovery was approximately −0.8 for both age classes, using Wishart priors resulted in median correlation estimates of −0.268 for juveniles and − 0.283 for adults. These posterior estimates appear to be well estimated as the posterior distributions are peaked and do not span the parameter space (Appendix S2) while substantially underestimating the magnitude of negative correlation such that 56% of these estimates for juveniles and 53% of these estimates of adults fell in the range $-0.3<\hat{\rho }<0.3$.

#### 3.2.3 Modest monitoring scenario

Our results did not meaningfully vary between constant and episodic monitoring scenarios in which cohort size was modest (Table 4), therefore we only summarize the modest scenario with constant cohort sizes of 250 juveniles and 800 adults in the text while presenting results for both modest scenarios (Table 4, Appendix S2). The median estimates of age‐specific correlation from models with Uniform priors fit to our modest data realizations $\left({\hat{\rho }}_{\mathrm{HY}}=-0.304,{\hat{\rho }}_{\mathrm{AHY}}=-0.577\right)$ were of substantially reduced magnitude relative to our reference values of $\rho_{R,\mathrm{HY}}=-0.801$ and $\rho_{R,\mathrm{AHY}}=-0.787$. Neither of these results is conclusive as the posterior distributions for most data realizations spanned the range of possible values for these parameters (Appendix S2). For example, 34% of the correlation estimates for juveniles fell in the range $\hat{\rho }<-0.7$ but another 20% fell in the range $-0.3<\hat{\rho }<0.3$ (Table 4), thereby providing similar levels of support to interpretation of strongly additive harvest and compensation. When models were fit with Gamma priors to these same modest and constant data realizations, median values were more negative $\left({\hat{\rho }}_{\mathrm{HY}}=-0.342,{\hat{\rho }}_{\mathrm{AHY}}=-0.865\right)$ when compared to Uniform priors, but these posterior distributions spanned the range of possible values and were bimodal (Appendix S2) such that 28% of the juvenile estimates and 10% of the adult estimates fell in the range $\hat{\rho }>0.3$. Ironically, the most conclusive results with modest data came from Wishart priors, but these results substantially underestimated correlation $\left({\hat{\rho }}_{\mathrm{HY}}=-0.105,{\hat{\rho }}_{\mathrm{AHY}}=-0.170\right)$ and were almost centered on 0 when correlation was strongly negative. The portion of these estimates falling in the range $-0.3<\hat{\rho }<0.3$ was 68% for juveniles and 62% for adults (Table 4). Unlike the correlation estimates from Uniform and Gamma priors, the combination of modest data and Wishart priors provided estimated correlation parameters that were peaked without spanning the parameter space, thereby not providing obvious evidence these data were inadequate (Appendix S2). If we were to adopt an approach of rejecting additive harvest on the criteria of the 95% credible interval overlapping 0, we would conclude harvest was compensatory 100% of the time with every modest data realization (Table 4, Appendix S2) even though correlation between survival and recovery was strongly negative.

## 4 DISCUSSION

After using previously published  methods for estimating and interpreting correlation between survival and recovery (e.g. Arnold et al., 2016), we advise against drawing strong conclusions for mallards given the inferential issues we uncovered. This is despite mallards being the most abundant duck species in North America and the waterfowl species with the most abundant tag‐recovery data. Particularly for juvenile females and adult males, the imprecision of these estimates precludes conclusions stronger than (1) correlation is more negative than not and (2) correlation is not strongly negative. Furthermore, our results depended on prior choice such that in the absence of a power analysis or comparison of different priors, we would not have any basis for concluding one prior to be more (or less) capable of recovering true parameters than another.

It is only through our power analysis that we could conclude that using Gamma priors will likely lead to overestimating the magnitude of negative correlation and Wishart priors likely underestimated the magnitude of negative correlation between survival and recovery. The discrepancies between results obtained with different priors and sample sizes highlight the potential for compromised or misleading inference from Bayesian analyses like ours when the model's behavior and power are not explored. Given the importance and interpretation applied to such correlation estimates in management and conservation contexts, we cannot overstate the potential for data limitations and prior choice, seemingly idiosyncratic modeling issues, to result in misleading inference that potentially leads to misguided harvest management recommendations.

With Wishart priors, we would infer female survival is more responsive to environmental conditions, and thereby less sensitive to harvest, when compared to results obtained using Gamma priors or Uniform priors (Figure 6). If annual survival is more variable and less precisely estimated with Wishart priors while annual recovery is insensitive to prior choice (Figure 6), then underestimating the magnitude of correlation with Wishart priors is an expected outcome relative to Uniform and Gamma priors. Reduced sensitivity of recovery estimates to prior choice is not entirely a surprise given the proximity of recovery estimates to the more‐precisely estimated boundary of the parameter space (Gelman et al., 2014) and the direct link between the data (recoveries) and recovery probability.

Overestimating the variability of survival and underestimating correlation with Wishart priors is consistent with results from Fay et al. (2021); these authors found heterogeneity among individuals was overestimated and correlation between traits underestimated with multivariate hierarchical models fit to capture–recapture data. The sensitivity of annual survival estimates to prior choice has implications beyond correlation analyses like ours to applications like sensitivity and elasticity analyses of vital rates with Bayesian‐integrated population models (Arnold, Clark, et al., 2017; Koons et al., 2017). Our results indicate that the contribution of survival to population growth could be over‐ or underestimated if the priors for tag‐recovery models within integrated population models led to over‐ or underestimating the variability of annual survival.

Our results also demonstrate that biological plausibility of parameters like mean survival and recovery (Table 3) does not ensure reliability of all the parameters estimated from a model, such as correlation between survival and recovery. Especially with our modest data realizations, mean survival estimates were biologically reasonable when corresponding estimates of correlation severely underestimated negative correlation between survival and recovery (Figure 7). The tendency of correlation estimates to span the parameter space when using models fit with Uniform or Gamma priors indicates data are insufficient to estimate random effects that do not overlap 0. This is problematic because similar sample sizes have been believed sufficient in several published analyses, but it appears reliable inference is not achievable with those modest sample sizes. Moreover, we are usually restricted to a single data set from which to draw inference and not the 50 realizations, such that the inadequacy of modest data for obtaining inference unclouded by sampling variability is not apparent when reviewing estimates of parameters like mean survival. While others have recognized the inadequacy of some tag‐recovery data for obtaining parameter estimates useful for informing waterfowl management (Sheaffer & Malecki, 1995), our findings about the impact of sample sizes on posterior inference is at least as important a result as the somewhat more expected influence of Bayesian priors. This is especially so because the sample sizes we found inadequate for detecting negative correlation have been thought more than adequate in recently published analyses despite previous cautions that these correlation analyses may have low power to detect negative correlation or additive mortality (Sedinger et al., 2010).

While power analyses or simulation studies should accompany complex empirical analyses, carefully assessing the support for multiple competing hypotheses applied to a posterior distribution (Wade, 2000) can also help avoid overconfident interpretation of results. In applications like ours, posterior distributions that span the parameter space or are bimodal (Appendix S2) simultaneously lend support to mutually exclusive ecological interpretations (Arnold, Afton, et al., 2017) (Table 4). For example, our results from modest data and Uniform priors could be interpreted as providing tentative support for moderate‐to‐strongly additive harvest, support for compensatory harvest (because the posterior distribution substantially overlaps 0), or inconclusive due to inadequate data (Appendix S2). We believe the latter interpretation—inadequate data—would be most appropriate.

The challenges we document in estimating correlation with accuracy are partially attributable to the sparsity of tag‐recovery data for species like mallards (or other waterfowl more generally). Our calculations of known‐fate years revealed that only 0.1 and 0.2 years of known‐fate data were obtained for every female and male mallard that was released with a band, respectively (Table 1). Given the relative sparsity of the female data, we focused our power analysis on female vital rates and sample sizes like those available for female mallards, northern pintail (Bartzen & Dufour, 2017), and lesser scaup (Arnold et al., 2016). Our example of 850 releases per year over 36 years provides a total sample of 28,800 adults (Table 2; AHY_850_). Of these adults, an average of 70–75 adults were known to be alive during each year of the study and our models relied on about 26 direct and about 33 indirect recoveries per year for parameter estimation (Table 2). These summary statistics emphasize the modest nature of these data sets, which may lead to less intrepid interpretations of parameters estimated from tag‐recovery data sets. We note that the efforts of the USGS Bird Banding Lab to increase reporting probability in the 1990s approximately doubled band‐reporting probability from an average of 0.3–0.4 to 0.7–0.8 (Arnold et al., 2020), thereby we expect statistical power per banded mallard to have doubled in years since 1996.

Instead of recommending these priors distributions (or others) for future correlation analyses or trying to explain why a prior works better for one analysis than another, we emphasize that variable prior influence among different priors is an expected outcome of Bayesian estimation. Careful consideration of the hypotheses associated with a prior and simulation may be the only way to avoid incorrect inference in some applications. In the case of the standard deviation of a random effect $\left(\sigma \right)$, trying to interpret this parameter may help understand why some priors are more useful than others. The hypothetical prior σ~0 (or an estimate of $\hat{\sigma }=0$) could be interpreted as the observed data and truth being the same. Similarly, the hypothetical prior σ~∞ (or an estimate $\hat{\sigma }=\infty$) could be interpreted as the data being of such extreme variability that they cannot be used to approximate truth. If we implement models with priors closer to σ~∞ (the Wishart prior), then correlation estimates tending toward 0 are an expected outcome; two parameters estimated from a prior hypothesizing infinitely variable data should not be expected to be highly correlated. Further, prior influence should be expected to be greater when data are relatively sparse (Gelman et al., 2014) and even more so when working with presence–absence data instead of continuous data (Fay et al., 2021).

While there are more circumstances that impact Bayesian estimation beyond prior choice than we can consider here, two aspects of our results warrant brief mention. First, median correlation estimates from our power analysis were more negative for juveniles than adults (Table 4). At the same time, juvenile survival and recovery probabilities (Figure 3b) spanned a greater range of values than for adults (Figure 4b). The range of values occupied by estimated parameters relative to the uncertainty of these estimates should be an important consideration when assessing the feasibility of estimating nonzero correlations; as the true variation of two parameters increases relative to sampling variation, correlation between these parameters should be easier to detect. Second, our results indicate survival probabilities were sensitive to prior choice, while recovery probabilities were generally insensitive to prior choice (Figure 6). We predict that prior choice would be less important if, for example, we were focused on estimating correlation between juvenile and adult recovery. Additionally, we predict correlation between juvenile and adult recovery would be estimated with greater precision than correlation between juvenile and adult survival, which were estimated with less precision (Figure 6). Considerations like these highlight the need for simulation work that closely matches the circumstances of complex analyses of capture–recapture data (Fay et al., 2021; Riecke et al., 2019), including tag‐recovery data.

Our power analysis indicates previous correlation analyses between survival and recovery (Arnold, Afton, et al., 2017; Arnold et al., 2016; 2017; Bartzen & Dufour, 2017) applied ecological interpretations to results that may not be statistically robust due to unrecognized data insufficiency (Table 5). In both cases, these analyses are characterized by a variety of potential deficiencies that led us to suspect the power of these analyses should be reevaluated. Future Bayesian analyses applying multivariate hierarchical models to tag‐recovery data to assess the impact of harvest on population dynamics, including integrated population models with tag‐recovery models in the joint likelihood (Arnold, Clark, et al., 2017; Koons et al., 2017), should include clearer descriptions of methods and prior distributions. Similar studies should also demonstrate through simulation or power analyses that the ecological questions being assessed can be answered with the data and statistical methods employed. We suspect some of the concerns identified in this manuscript are more broadly applicable to datatypes and hierarchical models beyond tag recoveries and the specific model type we evaluated.

**TABLE 5:** Possible paths to incorrect inference when negative correlation between survival and recovery is intepreted as support for additive harvest mortality but may also result from confounding between liberalized harvest opportunity and increased natural mortality when density dependent regulation increases with population size (Sedinger & Herzog, 2012).

Statistical inference	Ecological process	
	Liberalized harvest and density dependence	Additive harvest
Insufficient power to detect negative correlation	Statistically incorrect Biologically irrelevant	Statistically incorrect Biologically irrelevant
Negative correlation detected	Statistically correct Biologically incorrect	Statistically correct Biologically correct

## AUTHOR CONTRIBUTIONS

**Cody E. Deane:** Conceptualization (lead); data curation (equal); formal analysis (lead); funding acquisition (equal); validation (lead); visualization (lead); writing – original draft (lead); writing – review and editing (lead). **Lindsay G. Carlson:** Data curation (equal); writing – original draft (supporting); writing – review and editing (supporting). **Curry J. Cunningham:** Conceptualization (supporting); writing – original draft (supporting); writing – review and editing (supporting). **Pat Doak:** Writing – original draft (supporting); writing – review and editing (supporting). **Knut Kielland:** Writing – original draft (supporting); writing – review and editing (supporting). **Greg A. Breed:** Formal analysis (equal); funding acquisition (equal); supervision (equal); validation (supporting); writing – original draft (equal); writing – review and editing (equal).

## CONFLICTS OF INTEREST STATEMENT

No conflict of interest to declare.

## Supporting information


Appendices S1‐S3.

## Data Availability

The organized data (public data from USGS Bird Banding Lab) and R code files that support the results of this study are available at Dryad. https://datadryad.org/stash/share/Vpl3oxfrDk_AOlkos32KnIuc0y‐2Rk7oSu5ONxiA6Yw
