# Supplementary material for: Prior choice and data requirements of Bayesian multivariate hierarchical models fit to tag‐recovery data: The need for power analyses
Source: Ecol Evol. 2023 Mar 26;13(3):e9847. doi: 10.1002/ece3.9847 (PMC10041078; doi:10.1002/ece3.9847)
Supplement: Supplementary file 1 — Appendices S1‐S3. [file ECE3-13-e9847-s001.pdf]

ECOLOGY AND EVOLUTION

APPENDIX S1

Prior choice and data requirements of Bayesian multivariate hierarchical models fit to tag-recovery data: the need for power analyses

Deane, C.E., L.G. Carlson, C.J. Cunningham, P. Doak, K. Kielland, and G.A. Breed.

Equation S1: Under the hypothesis of additive harvest mortality, when harvest probability (*h*) increases by 0.01, survival probability (*S*) will decrease by 0.01; with the generic equation *y*=*mx*+*b* and a situation when band reporting probability is 0.40 $(f = h \times 0.4)$, the relationship between survival and recovery is expected to be –2.5.

$$y = \frac{-(S_2 - S_1)}{(h_2 - h_1)} x + b$$

$$m = \frac{-1 \times (S_2 - S_1)}{0.4 \times (h_2 - h_1)}$$

$$m = \frac{-2.5 \times (S_2 - S_1)}{(f_2 - f_1)}$$

$$y = \frac{-2.5(S_2 - S_1)}{(f_2 - f_1)} x + b$$

ECOLOGY AND EVOLUTION

APPENDIX S2

Prior choice and data requirements of Bayesian multivariate hierarchical models fit to tag-recovery: the need for power analyses.

Deane, C.E., L.G. Carlson, C.J. Cunningham, P. Doak, K. Kielland, and G.A. Breed.

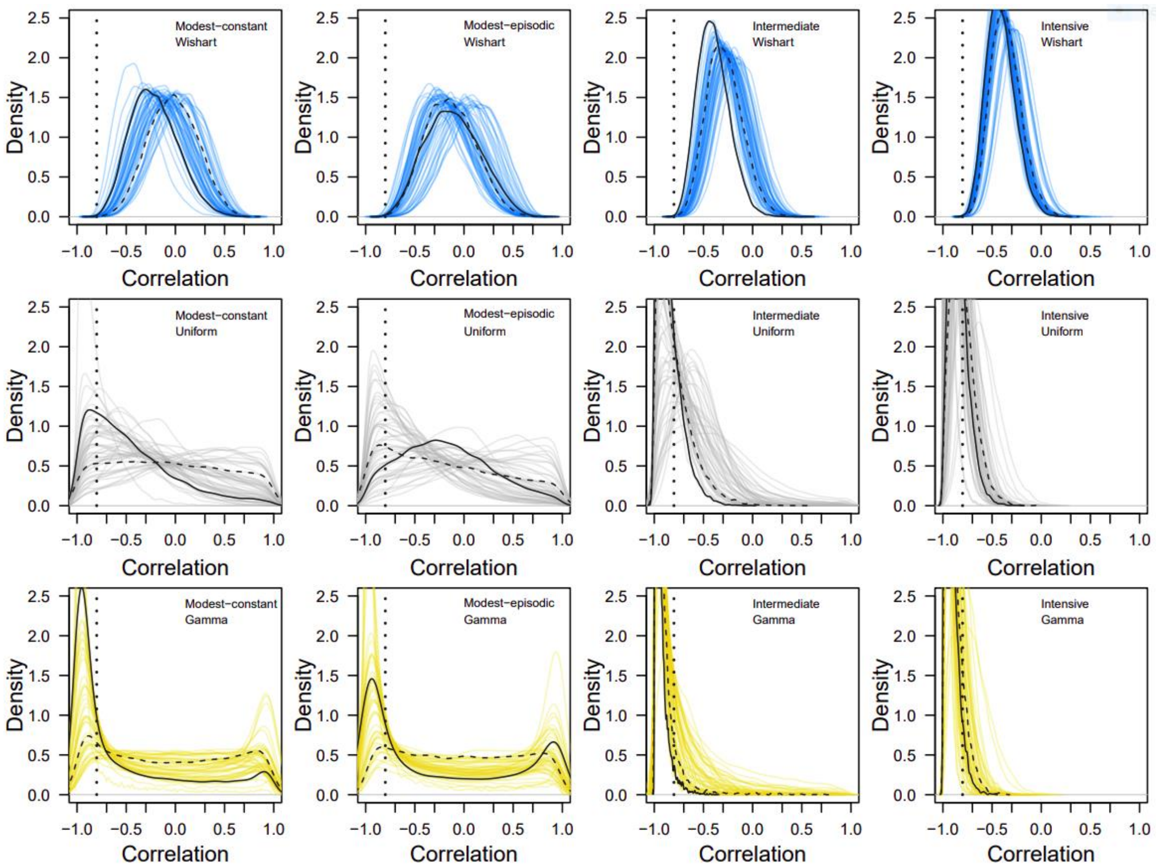

Figure S2–1. Posterior distributions of correlation between juvenile survival and recovery by prior distribution (rows) and monitoring scenario (columns). Each figure displays 50 posterior distributions, one for each data realization. Each column displays results for the same 50 data realizations. The correlation estimates for the first (solid line) and last (dashed line) data realization are highlighted, and the vertical line represents true correlation for the simulated population $(\rho_{R,HY} = -0.801)$.

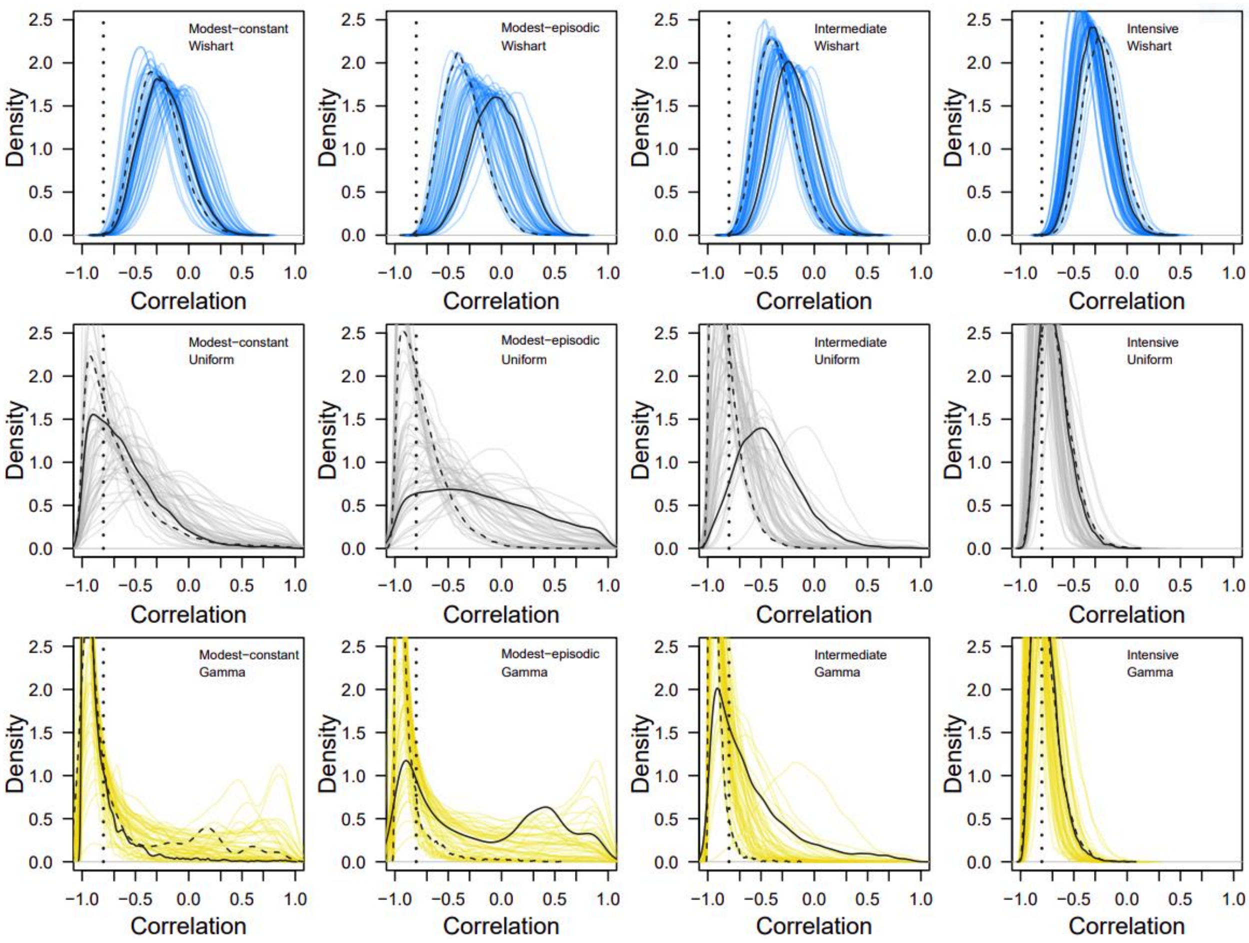

Figure S2–2. Posterior distributions of correlation between adult survival and recovery by prior distribution (rows) and monitoring scenario (columns). Each figure displays 50 posterior distributions, one for each data realization. Each column displays results for the same 50 data realizations. The correlation estimates for the first (solid line) and last (dashed line) data realization are highlighted, and the vertical line represents true correlation for the simulated population $(\rho_{R,AHY} = -0.787)$.

ECOLOGY AND EVOLUTION

APPENDIX S3

Prior choice and data requirements of Bayesian multivariate hierarchical models fit to tag-recovery: the need for power analyses.

Deane, C.E., L.G. Carlson, C.J. Cunningham, P. Doak, K. Kielland, and G.A. Breed.

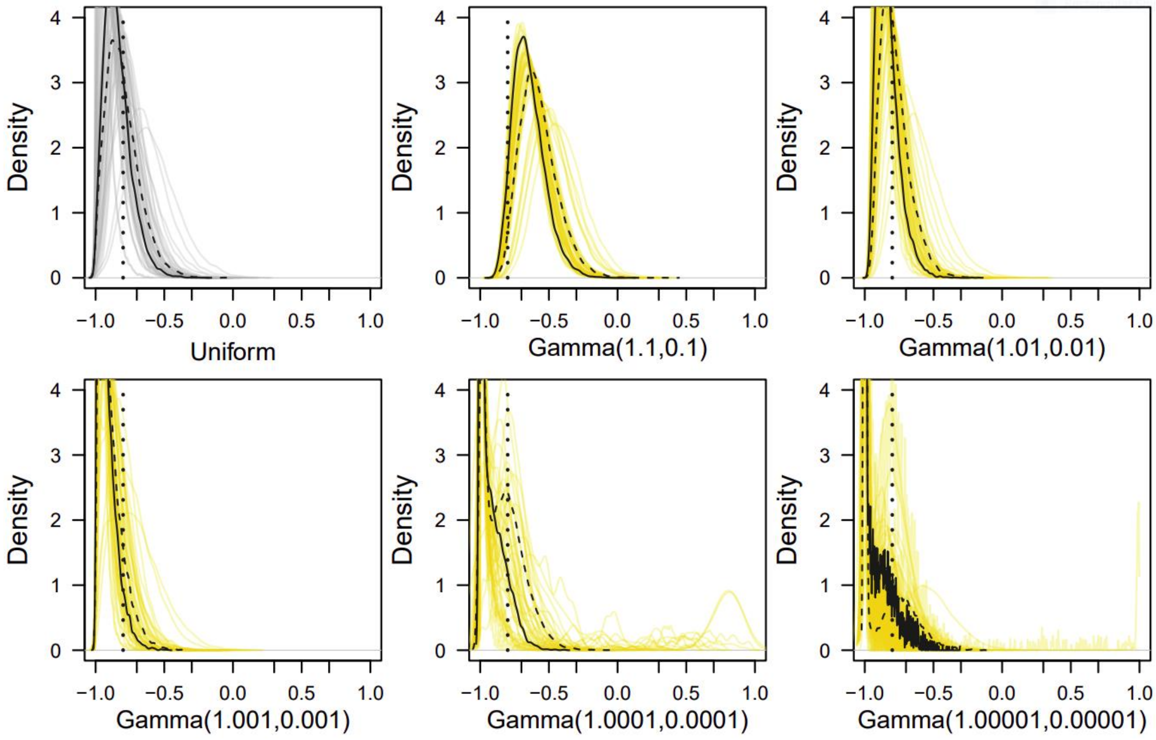

Figure S3–1. Posterior estimates of correlation between juvenile survival and juvenile recovery by prior distribution estimated from our intensive data realizations. Each figure displays 50 posterior distributions, one for each realization. Results vary by prior distribution, our Uniform prior and 5 formulations of a Gamma prior, and prior is displayed under the respective figure. The result for the first (solid line) and last (dashed line) data realization are highlighted, and the vertical line represents true correlation for our simulated population $(\rho_{R,HY} = -0.801)$.

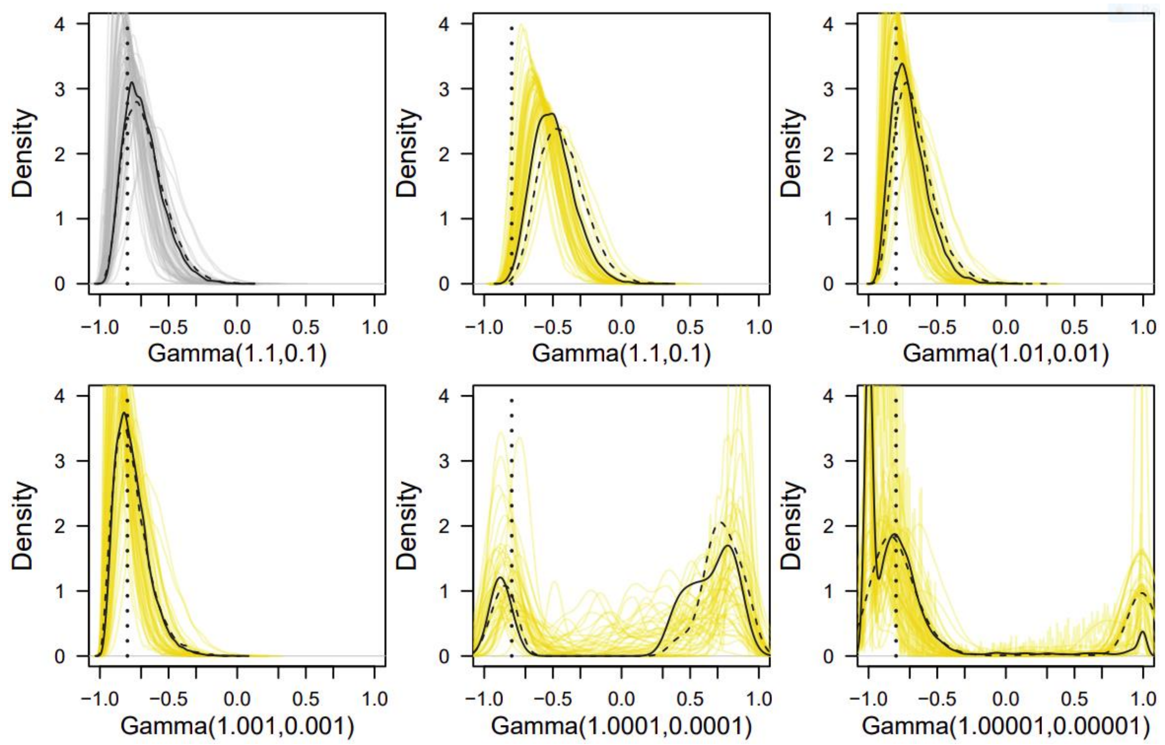

Figure S3–2. Posterior estimates of correlation between adult survival and juvenile recovery by prior distribution estimated from our intensive data realizations. Each figure displays 50 posterior distributions, one for each realization. Results vary by prior distribution, our Uniform prior and 5 formulations of a Gamma prior, and prior is displayed under the respective figure. The result for the first (solid line) and last (dashed line) data realization are highlighted, and the vertical line represents true correlation for our simulated population $(\rho_{R,AHY} = -0.787)$.
